# Qualitative evaluation of coronary atherosclerosis in a large cohort of young and middle-aged Dutch tissue donors implies that coronary thrombo-embolic manifestations are stochastic

**DOI:** 10.1371/journal.pone.0207943

**Published:** 2018-11-27

**Authors:** Jan H. Lindeman, Luuk Hulsbos, Antoon J. van den Bogaerdt, Marlieke Geerts, Alain J. van Gool, Jaap F. Hamming, Rogier A. van Dijk, Alexander F. Schaapherder

**Affiliations:** 1 Dept. of Surgery, Leiden University Medical Center, Leiden, The Netherlands; 2 Einthoven Laboratory for Experimental Vascular Medicine, Leiden University Medical Center, Leiden, The Netherlands; 3 Euro Heart Valve Bank, Beverwijk, The Netherlands; 4 TNO Metabolic Health Research, Leiden, The Netherlands; Stellenbosch University Faculty of Medicine and Health Sciences, SOUTH AFRICA

## Abstract

**Background and aims:**

With the intention to gain support for the hypothesis that incident ischemic complications of atherosclerotic disease involve a stochastic aspect, we performed a histological, qualitative evaluation of the epidemiology of coronary atherosclerotic disease in a cohort of aortic valve donors.

**Patients and methods:**

Donors (n = 695, median age 54, range 11–65 years) were dichotomized into a non-cardiovascular (non-CVD) and a cardiovascular disease death (CVD) group. Consecutive 5 mm proximal left coronary artery segments were Movat stained, and the atherosclerotic burden for each segment was graded (revised AHA-classification).

**Results:**

Non-CVD and CVD groups showed steep increase of atherosclerosis severity beyond the age of 40, resulting in an endemic presence of advanced atherosclerosis in men over 40 and women over 50 years. In fact, only 19% of the non-CVD and 6% of the CVD donors over 40 years were classified with a normal LCA or a so called non-progressive lesion type. Fibrous calcified plaques (FCP), the consolidated remnants of earlier ruptured lesions, dominated in both non-CVD and CVD donors. Estimates of the atherosclerosis burden (i.e. average lesion grade, proportion of FCPs, and average number of FCPs per cross-section) were all higher in the CVD group (*p*<1.10^−16^, *p*<0.0001, and *p*<0.05, respectively).

**Conclusions:**

Dominance of consolidated FCP lesions in males over 40 and females over 50 years, show that plaque ruptures in the left coronary artery are common. However, the majority of these ruptures remain asymptomatic. This implies that the atherosclerotic process is repetitive. A relative difference in disease burden between CVD and non-CVD donors supports the concept that complications of atherosclerotic disease involve a stochastic element.

## Introduction

Ischemic heart disease remains the leading cause of death worldwide.[1] While the successes of preventive life-style and medical interventions for ischemic heart disease are almost unprecedented, achievable risk reductions remain below 40%.[2] Moreover, it has been pointed out that the majority of ischemic events occur in persons currently not identified by risk profiling.[3,4] Although these reservations may imply shortcomings in current risk profiling models and management strategies, the high prevalence of residual disease may also reflect a degree of randomness in the development of the thrombo-embolic complications of coronary atherosclerosis (viz. ischemic heart disease being stochastic rather than deterministic).[5,6] This latter scenario is supported by plateauing of the C-statistics for cardiovascular risk prediction models at values between 0.75 and 0.80.[7–9]

Incident ischemic coronary events result from a complex sequence of events that involves atherosclerotic lesion formation and destabilization as the initial provocative factor. Actual development (or absence) of clinical manifestations relates to the occurrence or absence of thrombo-embolic complications. Development of thrombo-embolic complications and its clinical consequences reflect a complex interplay of pro- and anti-thrombotic factors, the fibrinolytic system, ischemia times, (residual) coronary lumen size, tissue oxygen demand, pre-existing collateral vascular networks etc.[10–12] In this context, we hypothesized that thrombo-embolic complications of coronary atherosclerosis are stochastically determined, and that human coronary atherosclerotic disease is a repetitive process that proceeds through multi-focal and asynchronous cycles of plaque initiation, -progression, and -destabilization. In absence of a fatal thrombo-embolic complication, plaque destabilization is followed by consolidation of the lesion.

The various aspects of the atherosclerotic process (viz. plaque initiation, -progression, and -destabilization followed by lesion consolidation) are best appreciated by histology as only this technique allows for assessment of the different stages of the disease. Discriminative plaque characteristics are captured by the AHA consensus classification scheme.[13,14] This scheme has been refined [15] (often referred to as the Virmani classification) in order to better mimic the natural history of the disease. More specifically, this revised classification scheme covers all distinct aspects of the atherosclerotic process (i.e. plaque initiation, -progression, and–destabilization, possible thrombo-embolic complications, as well as the aspects of plaque healing and scarring (lesion consolidation after destabilization (plaque rupture)).

We reasoned that if the assumption ‘that atherosclerosis reflects an asynchronous, repetitive process with thrombo-embolic complications as a stochastic (lethal) element’ is correct, this would translate in relative rather than in absolute differences in atherosclerotic lesion load in patients dying from cardiovascular causes and those from non-cardiovascular causes, with accrual of consolidated lesions [15] and a higher lesion burden [16] in the cardiovascular death group.

To test this hypothesis, we performed a systematic evaluation of the atherosclerotic load of the left main coronary artery, a primary predilection place for atherosclerotic disease, of young and middle-aged deceased Dutch tissue donors who donated their heart for aortic valve procurement. Two separate groups were created: one group dying from atherosclerosis-related (cardiovascular) causes, and a second group of donors who died from non-atherosclerotic related causes. The atherosclerosis load is evaluated and reported separately for each group.

## Methods

### Patients and tissue sampling

Use of the donor material for scientific purposes is approved by the Medical and Ethical Committee of the Leiden University Medical Center, The Netherlands. Sample collection and handling was performed in accordance with the guidelines of the Medical and Ethical Committee of the Leiden University Medical Center, and with the code of conduct of the Dutch Federation of Biomedical Scientific Societies. In all cases permission for transplantation orientated research was given or permission was inherent to donation.

Due to national regulations, only data relevant for transplantation was available for research (http://www.federa.org/codes-conduct). More specific information such as cholesterol or CRP levels were not available for the donors. Information on smoking (history) relied on hetero-anamnesis.

This study included data from 698 successive proximal left coronary artery (LCA) segments from as many Dutch donors who donated their heart for aortic valve procurement in the period 2011–2017. The age limit for aortic valve donation is 65 years, as such the maximum age of individuals in the study is 65 years. Further main exclusion criteria for donation include (a history of) malignancy, sepsis and/or risk of transferable disease (hepatitis, prions etc.), (suspected) connective tissue disorders or vasculitis/myocarditis. Moreover, donation criteria exclude men over 50 years with a history of diabetes, COPD or abdominal aortic aneurysm.

The LCA segments were collected during the aortic valve dissection. Valve procurement was performed within 40hrs after death at the Euro Heart Valve Bank (Beverwijk, The Netherlands). In short: the aortic valves were carefully removed from the intact heart, freed from the surrounding pericardial fat, and banked for transplantation. The surrounding pericardial fat tissue containing a segment of the proximal LCA (approximately 2-3cm) was formaldehyde fixed and used in this study. This collection procedure did not interfere with the actual valve dissection or the pathological analysis of the heart necessary for the final release of the banked valves.

Two study groups were created: a non-cardiovascular death (non-CVD) group and a cardiovascular death (CVD) group. The non-CVD group included all donors whose cause of death was not atherosclerosis-related (viz. suicide, high energy trauma, subarachnoid haemorrhage, subdural hematoma, venous thrombosis). The CVD group included all individuals who died from atherosclerosis-related causes (viz. myocardial infarction, cerebrovascular accident).

Histological lesion classification.

**Fig 1:**
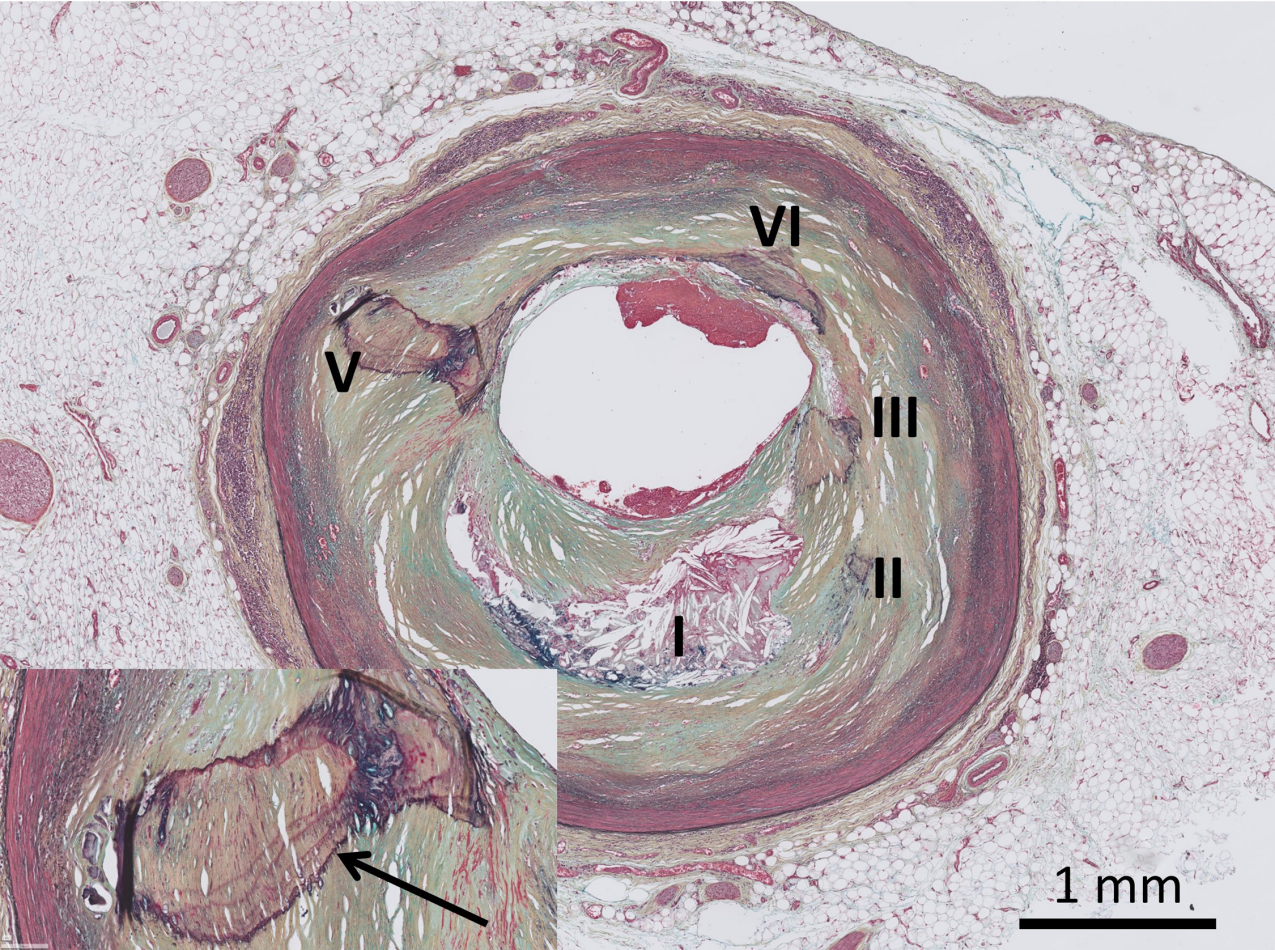
Movat staining of a coronary artery cross section illustrating the foot prints of consolidated calcified lesions (purple-brown demarcations indicated by the arrow in the enlarged insert), and the presence of multiple lesions within a single cross section. I: late fibro-atheroma (LFA) lesion (necrotic core covered by a fibrous cap). II-V: indicating consolidated former lesions (fibrotic calcified plaque (FCP)) [15].

The formaldehyde-fixed tissue was decalcified (Kristensen's solution) in order to facilitate proper sectioning. Decalcification does not interfere with (calcium) scoring, as histological footprints of earlier calcium deposits remain present after the process of decalcification (brown and dark purple deposits in Movat staining illustrated in Fig 1).

**Fig 2:**
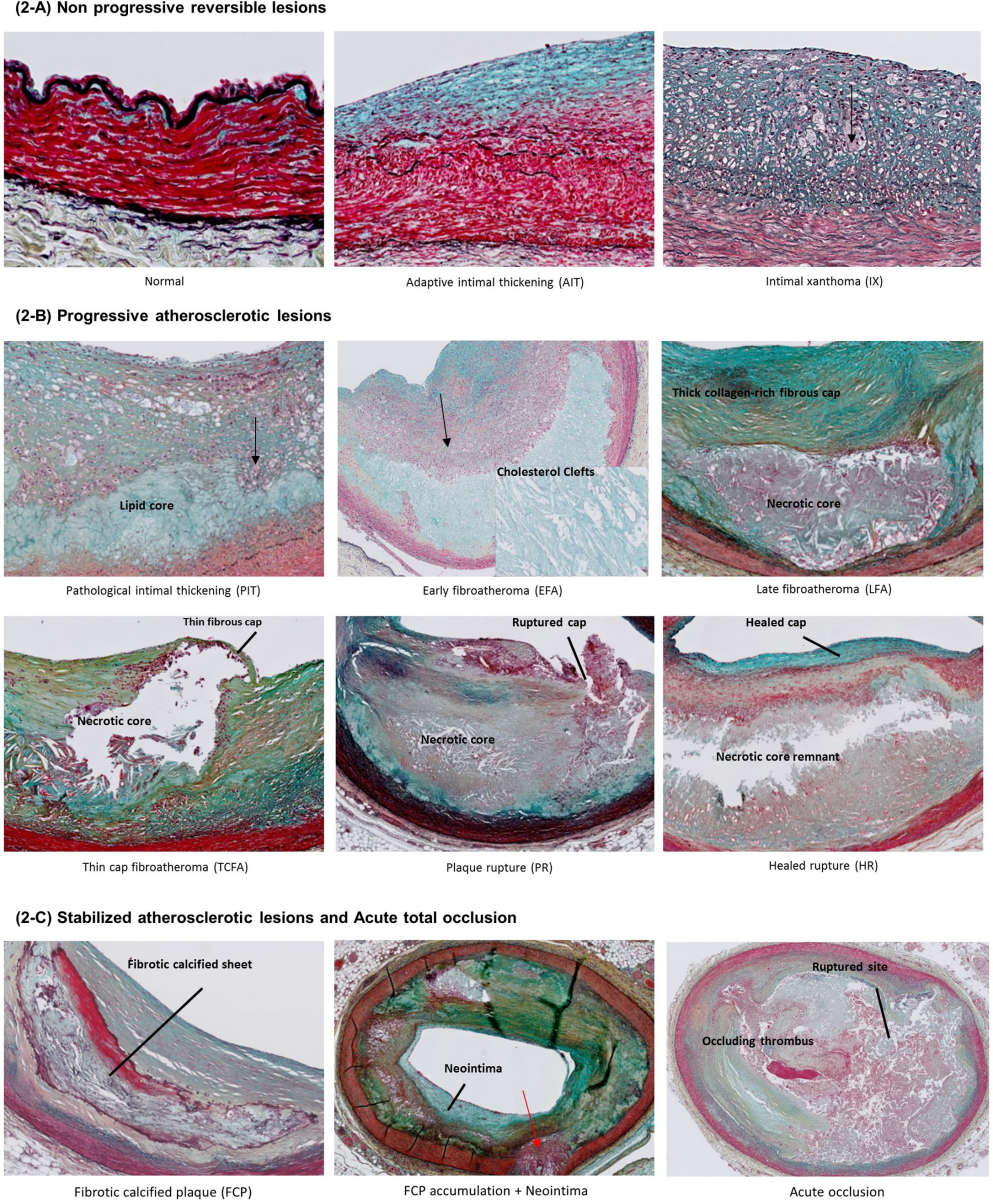
Adapted AHA (Virmani) classification of coronary atherosclerotic lesions [15] (Movat stained coronary artery segments). (2-A) Normal and non-progressive (reversible) lesions [15] Adaptive intimal thickening (AIT) is characterized by hyperplasia of the tunica intima (blue); intimal infiltration of macrophages (black arrows) and presence of foam cells characterize intimal xanthoma (IX). (2-B) Progressive atherosclerotic lesions.[15] Pathological intimal thickening (PIT) is hallmarked by a pre-necrotic lipid core, with or without surrounding infiltrated foam cells (black arrows). Early and late fibro-atheroma (EFA and LFA) are characterized by a necrotic lipid core, cholesterol crystals, and an overlying thick collagen-rich (yellow/green) fibrous cap. Thin cap fibro-atheroma (TCFA) is characterized by a thin fibrous cap, which precedes rupture (plaque rupture (PR)). Rupture is followed by a healing process with formation of a new proteoglycan/cell rich cap (healed rupture (HR)). (2-C) Stabilized atherosclerotic lesions and acute total occlusion.[15,16] The healed rupture ultimately transforms into a scar, the fibrotic calcified plaque (FCP) contains calcified remnants of the necrotic core. New lesions can develop on top of FCPs (neo-intima, blue), which can ultimately result in stacked lesions. This may eventually cause accumulation of multiple lesions within one cross section (red arrows) and formation of a neo-intima overlying the consolidated earlier lesions. Acute occlusion represents an example of a (fatal) thrombo-embolic acute full occlusion of the LCA.

Coronary arteries were divided in consecutive 5mm segments. Each segment was paraffin embedded, and 4μm sections were prepared for each individual segment. Movat pentachrome staining was performed for each 5mm segment, and all segments were classified (revised classification of the American Heart Association (AHA) as proposed by Virmani et al. [15], Table 1 and Fig 2). Classification was performed by at least two experienced observers with no knowledge of the donor characteristics. Incongruent opinions were resolved in separate sessions with a third attending observer.

**Table 1:** Adapted AHA classification schema for human atherosclerosis [15].

Normal (Norm)	Thin intima, minimal presence of smooth muscle cells
Adaptive Intimal Thickening (AIT)	Thickening of the intima, smooth muscle cells crossed the internal elastic lamina
Intima Xanthoma (IX)	Adaptive Intimal Thickening + presence of foam cells
Pathological Intimal Thickening (PIT)	Thickened intima with a structured lipid core
Early Fibro-atheroma (EFA)	Thickened intima with a structured lipid core and small cholesterol crystals
Late Fibro-atheroma (LFA)	Large confluent lipid core with large cholesterol crystals that is covered by a thick collagenous cap
Thin Cap Fibro-atheroma (TCFA)	Large confluent lipid core with large cholesterol crystals that is covered by a thin (<65μm) collagenous cap
Ruptured Plaque (RP)	Discontinuation (rupture) of the collagenous cap
Healed Rupture (HR)	Coverage of the rupture lesion with proteoglycan/smooth muscle cell-rich matrix
Fibrous Calcified Plaque (FCP)	A fibrotic, a-cellular lesion with one or multiple condensed, calcified remnants of a necrotic core

For each donor, the section of the tissue segment showing the most advanced lesion type was used in this study (reference-section). Many donors presented with multiple consolidated end-stage lesions (fibrous calcified plaques (FCPs)) in the reference-section. These sections were further sub-classified on basis of the sum of separate FCP lesions present in the reference-section (illustrated in Fig 1). Chronic occlusion [15, 16] was defined as coronary arteries showing a full fibrotic occlusion of the lumen.

### Statistical analysis

Statistical analysis was performed using SPSS 22 (IBM, Amsterdam, The Netherlands). The Virmani atherosclerosis grading [15] used is a descriptive progressive score. In order to allow statistical evaluation of atherosclerosis progression and comparison of atherosclerosis burden we reclassified the lesions types (Fig 2A–2C) in a progressive numeric score (normal = 1, adaptive intimal thickening = 2, intima xanthoma = 3…, one FCP lesion per cross section = 10, 2 FCP lesions per cross-section = 11, 3 FCP lesions per cross-section = 12, 4 FCP lesions per cross-section = 13, 5 FCP lesions per cross-section = 14, chronic occlusion = 15). Numeric scores were considered as a continuous variable, and associations conservatively estimated through generalized estimating equations. P values below 0.05 were considered significant.

In order to avoid interpretation problems due to over-plotting, data points in the graphs have been jittered. [17] Individual data is available in the supplemental data sheet.

## Results

This study includes data for the proximal LCA of 698 aortic valve donors (376 men and 322 women). The 65-year upper age limit was dictated by the eligibility criteria for valve donation. Donors were sub-classified on basis of their cause of death in a non-CVD group (n = 335) and a group that died from atherosclerosis-related causes (CVD group, n = 363). Reportedly, approximate 24% of all deaths (46.7% in the CVD group) were related to an acute myocardial infarction. Three patients in the CVD-death group had an intracoronary stent in their LCA, and could therefore not be graded. These individuals were excluded from the analysis.

Baseline characteristics for the two groups are provided in Table 2. Since young age is a major confounder of cause-of-death (i.e. cardiovascular death is rare before the age of 35, and young males dying from unnatural causes dominate in the younger aged deaths), characteristics for individuals over 40 are also presented separately in Table 3.

**Table 2:** Donor characteristics for the full cohort (A)). (mean [sd] or absolute number (proportion)). P-values are for the non-CVD† vs. CVD cohorts.

	Non CVD †	CVD †	
N	335	360	
Sex (male)	44.8%	62.7%	p<0.0001
Age (Years)	48.0 [13.7]	54.21 [7.5]	p<1 10^−15^
BMI (kg/m2)	24.8 [3.9]	25.9 [3.1]	p<0.0001
Cause of death			
Trauma	98 (27.2%)	-	
Asphyxia	81 (24.2%)	-	
Pulmonary Embolism/ Dissection	42 (12.5%)	-	
Sub Arachnoid- or Subdural Hematoma	91 (27.2%)	-	
Metabolic	23 (6.9%)	-	
Myocardial Infarction	-	163 (46.7%)	
Cerebral Vascular Accident	-	194 (54.3%)	

**Table 3:** Donor characteristics for the individuals† over 40 years of age. (mean [sd] or absolute number (proportion)). P-values are for the non-CVD vs. CVD cohorts.

N	249	350		
Sex (male)	39.4%	62.6%		p<1.10^−7^
BMI (kg/m2)	25.1 [4.0]	25.9 [3.1]		p<0.01
(Treated) Hypertension	78 (25.5%)	125 (35.6%)		NS
Statin use	21 (7%)	42 (12%)		NS
% individuals with non- progressive lesions	19.7%	6.3%		p<0.0001
Mean lesion grade*)	6.23 (3.3)	8.67 (3.0)		p<1.10^−16^
% individuals with consolidated lesion(s) present	35.0%	65.1%		p<0.0001
Mean # consolidated lesions	1.44 [0.68]	1.63 [1.00]		p<0.05
Mean age of patients with one or more consolidated lesions				
Males	55.3 (6.1)	54.9 (5.0)		NS
Females**	59.3 (6.3)	58.2 (6.5)		NS

† CVD: cardiovascular death

**Fig 3:**
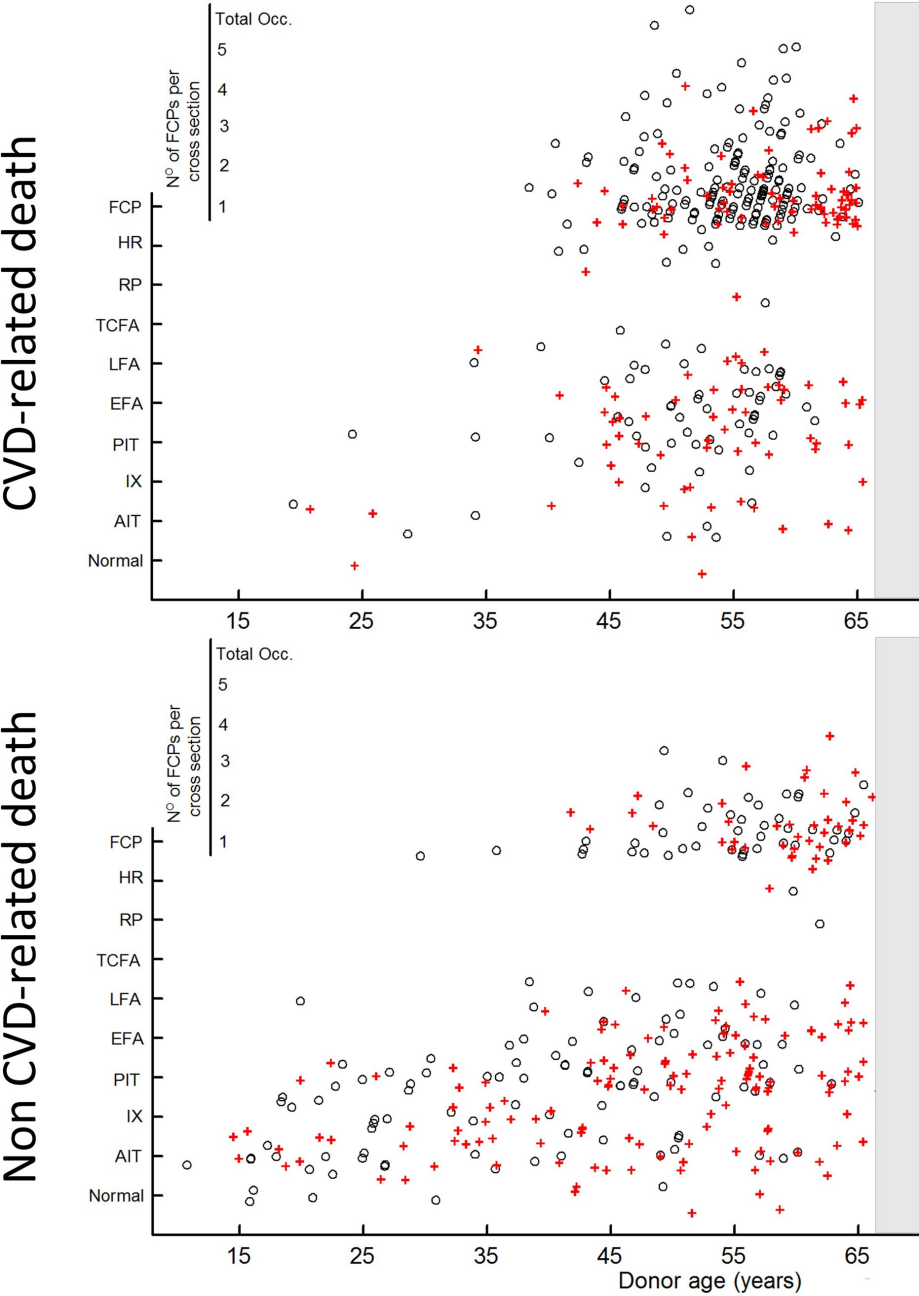
Age distribution for the most advanced lesion type present in the proximal left coronary artery .segment studied. Fig 3A: non-cardiovascular death donors. Fig 3B: cardiovascular death donors. ‘+’ represent females and ‘o’ represent males. Data points have been jittered [17] in order to avoid overlap.

*) lesion types in the Virmani classification [15, 16] were reclassified in a progressive numeric score (normal = 1…, 1 FCP in the cross section = 10, 2 FCPs = 11 etc., chronic occlusion = 15). Individual lesion grading for all cases including individuals <40 years of age is shown in Fig 3.

**) Females with at least one consolidated lesion significantly older than the males: non CVD group: P<0.004; CVD group: P<0.0003.

In those over 40, the data for the CVD group showed a clear dominance of male sex (*p*<1.10^−7^) and a slightly higher BMI (*p*< 0.02). Proportions of individuals treated for hypercholesterolemia and/or hypertension were similar in the CVD and non-CVD group.

The age distribution of atherosclerosis (defined by most advanced lesion type present (grading summarized in Table 1 and illustrated in Fig 2)) for all individual LCAs is shown in Fig 3A (non-CVD individuals) and Fig 3B (CVD individuals). Qualitative grading showed an endemic presence of non-progressive atherosclerotic lesions (AIT and IX) and pathological intimal thickening (Fig 3A and 3B) in individuals under 30. Advanced lesions were absent in the younger donors.

For both groups, the age of 40 marks a transition after which the more advanced lesion types become prominent (Fig 3A and 3B). This resulted in an endemic presence of progressive atherosclerotic lesions in individuals over the age of 40 years. In fact, over the age of 40 only 19.1% (95% CI: 14.7–24.5%) in the non-CVD and 6.0% (95% CI: 4.0–9.0%) in the CVD group was classified as having a normal LCA or a LCA with a non-progressive atherosclerotic lesion. [15] Moreover, the consolidated (end-stage atherosclerotic disease) fibrous-calcified plaque (FCP) lesion-type was absent in donors under 35, but was by far the most prevalent lesion type in individuals aged 40 and over. In fact, the LCA of 65.1% (95% CI: 59.9–70.3%) of the CVD donors and 35.0% (95% CI: 29.1–41.3%, *p*<0.0001) of the non-CVD donors was classified with a FCP in the reference cross-section. Many donors presented with multiple FCP lesions in the reference cross-section (Fig 1). The number of individual FCP lesions in the reference cross-section of each donor is represented in Fig 3A and 3B. Total occlusion of the LCA was observed in three donors: one acute fatal thrombotic total occlusion, and a chronic (fibrotic, *viz*. non-lethal total occlusion) in two donors. The prevalence of so-called vulnerable (culprit) lesions (*i*.*e*. thin cap fibro-atheroma (TCFA), ruptured plaque (RP) or healed rupture (HR)) was 3.5% (95% CI: 1.8–6.3%) in men and 1.6% (95% CI: 0.6–3.9%) in women over 40.

## Discussion

This systematic, qualitative evaluation of coronary atherosclerosis burden of the proximal left coronary artery of young and middle-aged tissue donors shows a relative rather than an absolute difference in disease burden between individuals dying from cardiovascular- and those dying from non-cardiovascular causes. Accumulation of consolidated FCP lesions in men over 40 and women over 45 implies that incident plaque rupture, and subsequent healing and scarring is a common phenomenon.

This comparative qualitative study is performed on material from a unique and large biobank of proximal LCAs from (deceased) Dutch donors who donated their heart for aortic valve replacement procedures. The LCA is an established predilection place for (coronary) atherosclerosis. In fact, intravascular ultrasound studies show a mild gradient with the higher ‘plaque burden’ for the more proximal aspects of the coronary arterial tree. [18, 19]

Multiple studies have shown that the manifestations and complications of atherosclerotic disease essentially relate to the qualitative rather than quantitative (i.e. degree of occlusion) aspects of the process. [20, 21] Accordingly, we applied a systematic histological evaluation (‘grading’) as only this technique allows for a full appreciation of *qualitative* aspects of the atherosclerotic process. [15] More specifically, atherosclerosis grading was performed for every 5 mm segment of each LCA using the Virmani classification. [15] This modification of the AHA classification^14^ better incorporates the natural history of the atherosclerotic process; viz. plaque initiation, lesion (lipid core) formation and -progression, plaque destabilization, and ultimate rupture. In the absence of fatal complications, rupture is followed by plaque healing and lesion consolidation. [15, 16] Consolidated lesions are characterized by a condensed, often calcified remnant of the original lipid core, and a surrounding, a-cellular collagenous (fibrotic) matrix; the ‘fibrous calcified plaque’ as described in the Virmani classification. [15]

Although the data from this evaluation confirms observations from earlier quantitative reports showing that atherosclerosis manifests early in life, [22–28] this qualitative analysis shows for young individuals (i.e. age below 40 years) that the process remains essentially limited to early lesion types (adaptive intimal thickening, intima xanthoma and pathological intimal thickening), and that the more advanced lesions types are absent in young individuals. Absence of advanced lesion types in young individuals in this cross-sectional study supports the notion that the early lesion types can be non-progressive, yet finite conclusions can obviously only be drawn from prospective follow up studies.

The age of 40 years marks a critical tipping point beyond which advanced atherosclerotic lesion types become endemic. In fact, this study indicates dominance of the FCP lesion types [15, 16] in the individuals dying from cardiovascular causes and, to a somewhat lesser extent, in those dying from non-cardiovascular causes. Although this phenomenon obviously reflects the stable, presumable (semi) permanent nature of these lesions, it also signals consolidation (healing) of an earlier non-fatal plaque rupture. [15, 16] As such, the data implies that plaque ruptures are rather common in individuals over 40, and often pass unnoticed.

The higher FCP load in CVD-death group supports the concept that thrombo-embolic complications of coronary atherosclerosis involve a stochastic element. That is to say, each bout of plaque rupture comes with a risk for a threatening thrombo-embolic complication, and consequently the more plaque ruptures, the higher the chance of a fatal life-threatening event. A further noteworthy observation was the common presence of multiple adjacent, or even stacked FCP lesions in a single LCA cross-section. This observation implies that atherosclerosis is a repetitive process.

The data further indicates a low prevalence of classic, advanced atherosclerotic lesion types (EFA and LFA) and vulnerable lesions. [15, 16] This implies that the process of atheroma formation, -progression, and lesion destabilization is relatively rapid. This observation, that along with the apparent repetitive character of the atherosclerotic process, and the putative stochastic aspects of thrombo-embolic complications provide a rationale for the current focus shift from plaque vulnerability to plaque burden as a superior ‘predictor’ for future ischemic events. [29] Although the plaque burden itself does not relate to future ischemic events, it does reflect the individual’s past and consequently the odds for a future event. [30]

It came to our attention that the apparent repetitive nature of the atherosclerotic process with asynchronous cycles of atherosclerotic lesion initiation and evolution provides a rationale for the observed delays in benefit reported for clinical cardiovascular risk management. [31, 32] In fact, both studies on lipid lowering (statins) [31] as well as those targeting inflammation (Cantos trial) [32] show an approximate 12-month delay before a therapeutic benefit becomes apparent. One could speculate that the main benefit of these interventions is a reduction in lesion formation rather than on lesion progression and destabilization.

Limitations: this study is based on the proximal LCA segment collected during aortic valve procurement for tissue donation. Consequently, information on other segments of the coronary arterial tree is missing. The proportion of patients dying from stroke is higher than expected, [33] an observation that is presumably secondary to the criteria for heart valve donation. As result of the capped age limit in donation guidelines, this study only includes data from individuals dying before the age of 65. As such, results may not be representative for the elderly population. Moreover, material in this biobank is from individuals who died from sudden death. A large proportion of whom without a medical history, hence without information on cardiovascular risk factors including genetic predisposition. In those with a medical history, only data for medication use and BMI was available. Available data on blood pressure is influenced by the medical condition leading up to death. Lipid profiles and a reliable smoking history are not available. A more in-depth evaluation of those on pharmaceutical cardiovascular risk management (anti-hypertensives and/or cholesterol lowering) is obviously obscured by the varying levels of pharmaceutical cardiovascular risk management, and by confounding-by-indication. As such no conclusions can be drawn from these parameters.

For the younger individuals, a further bias may result from clinical competition between tissue (valve), and organ (heart transplant) donation for the ‘healthier’ donors, and thus that LCAs from healthier young donors are underrepresented. This will obviously not impact the core conclusions of this study as these are essentially based on information from the donors over the age of 40 years. Moreover, we consider the potential impact minimal as we observed remarkable parallels between the atherosclerosis distribution in this LCA cohort, and our earlier published data for the peri-renal artery aorta (a predilection location for human atherosclerosis). [34] Material for this evaluation was collected during organ procurement for kidney transplantation, a context without competition between organ- and tissue donation, and less stringent age restrictions.

In conclusion, this cohort of Dutch tissue donors shows that advanced atherosclerotic disease is endemic over the age of 40 (men) and 45 (women). Asymptomatic plaque ruptures appear common, supporting the concept that thrombotic-embolic complications after plaque rupture reflect a stochastic event.

## Supporting information

S1 Table Individual data. Lindeman et al Qualitative evaluation of coronary dataset.
(XLS)

## Abbreviations

non CVD: non-cardiovascular death
CVD: cardiovascular death
LCA: left coronary artery
